# CNV discovery for milk composition traits in dairy cattle using whole genome resequencing

**DOI:** 10.1186/s12864-017-3636-3

**Published:** 2017-03-29

**Authors:** Yahui Gao, Jianping Jiang, Shaohua Yang, Yali Hou, George E Liu, Shengli Zhang, Qin Zhang, Dongxiao Sun

**Affiliations:** 1 Key Laboratory of Animal Genetics and Breeding of Ministry of Agriculture, National Engineering Laboratory of Animal Breeding, College of Animal Science and Technology, China Agricultural University, Beijing, 100193 China; 2 CAS Key Laboratory of Genomic and Precision Medicine, Beijing Institute of Genomics, Chinese Academy of Sciences, Beijing, 100101 China; 3 Animal Genomics and Improvement Laboratory, BARC, USDA-ARS, Beltsville, Md 20705 USA

**Keywords:** Copy number variation, Chinese Holstein, Whole genome re-sequencing

## Abstract

**Background:**

Copy number variations (CNVs) are important and widely distributed in the genome. CNV detection opens a new avenue for exploring genes associated with complex traits in humans, animals and plants. Herein, we present a genome-wide assessment of CNVs that are potentially associated with milk composition traits in dairy cattle.

**Results:**

In this study, CNVs were detected based on whole genome re-sequencing data of eight Holstein bulls from four half- and/or full-sib families, with extremely high and low estimated breeding values (EBVs) of milk protein percentage and fat percentage. The range of coverage depth per individual was 8.2–11.9×. Using CNVnator, we identified a total of 14,821 CNVs, including 5025 duplications and 9796 deletions. Among them, 487 differential CNV regions (CNVRs) comprising ~8.23 Mb of the cattle genome were observed between the high and low groups. Annotation of these differential CNVRs were performed based on the cattle genome reference assembly (UMD3.1) and totally 235 functional genes were found within the CNVRs. By Gene Ontology and KEGG pathway analyses, we found that genes were significantly enriched for specific biological functions related to protein and lipid metabolism, insulin/IGF pathway-protein kinase B signaling cascade, prolactin signaling pathway and AMPK signaling pathways. These genes included *INS, IGF2, FOXO3, TH, SCD5, GALNT18, GALNT16, ART3, SNCA* and *WNT7A*, implying their potential association with milk protein and fat traits. In addition, 95 CNVRs were overlapped with 75 known QTLs that are associated with milk protein and fat traits of dairy cattle (Cattle QTLdb).

**Conclusions:**

In conclusion, based on NGS of 8 Holstein bulls with extremely high and low EBVs for milk PP and FP, we identified a total of 14,821 CNVs, 487 differential CNVRs between groups, and 10 genes, which were suggested as promising candidate genes for milk protein and fat traits.

**Electronic supplementary material:**

The online version of this article (doi:10.1186/s12864-017-3636-3) contains supplementary material, which is available to authorized users.

## Background

In dairy cattle, milk yield and composition are the most important economic traits. Compared with traditional dairy cattle breeding programs, DNA-based marker-assisted selection has obvious advantages to shorten generation interval and enhance the accuracy of selection. There are several main strategies of identifying the genes with large genetic effects on milk production traits, including marker-QTL linkage analysis (LA), candidate gene approach, genome-wide association analysis (GWAS) and next-generation sequencing (NGS). Many studies have been performed to investigate milk production traits in dairy cattle [1–15].

Copy number variations (CNVs) are DNA segments that are 1 kb or larger and present at variable copy number in comparison with a reference genome [16]. CNVs are widely distributed in the genome [17]. As a complementary genetic variant to single nucleotide polymorphisms (SNPs), CNVs have attracted increasing attention in recent years. Compared with the SNP, CNV can affect a larger portion of the genome and cause effects, like changing gene structure and dosage, altering gene regulation and exposing recessive alleles [18]. CNVs are also associated with various diseases [19, 20] and may contribute to a fraction of the missing heritability [21, 22]. Along with the development of large-scale CNV studies in human [16], substantial progress has been made in the CNV identification in domestic animals, including cattle [13, 23–34], dog [35–37], sheep [38], goat [39], chicken [40, 41] and pig [42–44]. So far, there are four mechanisms known to allow formation of CNV, i.e., non-allelic homologous recombination (NAHR), non-homologous end joining (NHEJ), fork stalling and template switching (FoSTeS) and retrotransposition [18, 45]. In addition, previous studies also suggested that segmental duplications (SDs) are one of the catalysts and hotspots for CNV formation [46, 47].

Traditionally, there were two array-based methods for CNV discovery, array comparative genomic hybridization (aCGH) and SNP arrays [48, 49]. Although they promoted the progress of CNV studies, these two array-based methods have limitations [50, 51]. They cannot detect small CNVs [52]. In addition, it is also a great challenge for microarrays to detect CNVs in the SD regions due to insufficient coverage [53] although customized chips can be designed to cover SDs.

Recently, the advent of next-generation sequencing (NGS) technology has sped up the study of CNV [23, 27, 28, 54]. Four basic strategies have been applied for detecting CNVs with NGS data in the 1000 Genomes Project pilot studies [55]. Read pair (RP or paired-end mapping) method [56, 57] analyzes discordant mapping pairs of clone reads or high-throughput sequencing fragments whose distances are different from the normal average insert size. Read-depth (RD) [58–60] analysis detects CNVs based on the read depth-of-coverage, i.e., the density of aligned reads along the chromosomes. A random distribution (Poisson distribution or corrected Poisson distribution) is assumed first in this method. Based on the depth-of-coverage, CNVs are detected with duplication regions showing high coverage, while deletion regions show low coverage. Split-read (SR) [59, 61] analysis can evaluate gapped sequence alignments for CNV detection. This method first splits one read into multiple fragments randomly. Then the first and last fragment aligned along the reference genome respectively. According to whether the fragments align or not, and the locations and directions if aligned, CNVs can be detected. The mechanism of SR is similar to RP to some extent. Sequence assembly (AS) method [62, 63] could discover all kinds of genetic variations theoretically because of its fine-scale working. The direct assembly of short reads without reference genome is called *de novo* assembly and the general strategy is to reconstruct DNA fragments, i.e., contigs, based on assembling overlapping reads firstly. Then by comparing the assembled fragments to the reference genome, the abnormal genomic regions with discordant copy number (CN) can be identified. Additionally, AS-based methods can also use a reference genome to improve the computational efficiency and contig quality.

RD methods applied in the 1000 Genomes Project data have been shown to predict accurate copy number values due to its capability of high-resolution CNV calls [19]. There have been several approaches based on RD, such as MAQ [52, 64], SegSeq [58], mrFAST [47] and CNVnator [65]. CNVnator can overcome some disadvantages, including unique regions of the genome [52, 58, 64], poor breakpoint resolution [47, 52, 58, 64], and detect different sizes of CNVs, from a few hundred bases to megabases in the whole genome. For CNVs accessible by RD described Abyzov et al. [65], CNVnator has high sensitivity (86 ~ 96%), low false-discovery rate (3% ~ 20%), high genotyping accuracy (93% ~ 95%), and high resolution in breakpoint discovery. In addition, they estimated that at least 11% of all CNV loci involve complex, multi-allelic events, a considerably higher estimate than reported earlier [66].

For the CNV detection in the cattle genome, there have been several studies reported using such methods, including CGH [67, 68], BovineSNP50 Beadchip [32, 69, 70], BovineHD SNP Beadchip [25, 31] and NGS [23, 27–30]. In this study, the objective was to identify candidate genes for milk protein and fat traits of dairy cattle through CNV detection based on NGS data of specific Holstein bulls that have extremely high and low estimated breeding values (EBVs) for milk protein and fat percentages.

## Methods

### Animals and re-sequencing

Eight proven Holstein bulls with high reliabilities (>0.90) of estimated breeding values (EBVs) for milk protein percentage (PP) and fat percentage (FP), born between 1993 and 1996, were selected from the Beijing Dairy Cattle Center (http://www.bdcc.com.cn/) according to their EBVs for PP and FP. EBVs were calculated based on a multiple trait random regression test-day model using the software RUNGE by the Dairy Data Center of China (http://www.holstein.org.cn/). The bulls were from two half sib families and two full sib families with two bulls in each family. The two bulls in each group showed extremely high and low EBV for milk PP and FP, respectively. The detailed information of the 8 bulls is present in Table 1.

**Table 1:** The estimated breeding values and family information about 8 Holstein bulls

Family	Sample	Relationship	EBV for PP	EBV for FP	Reliability
1	1	Full-sib	0.03	0.1	0.99
	2		−0.13	−0.31	0.97
2	3	Full-sib	−0.03	0.27	0.98
	4		0.08	0.56	0.99
3	5	Half-sib	0.01	−0.26	0.99
	6		0.22	0.09	0.91
4	7	Half-sib	0.07	−0.14	0.98
	8		−0.06	−0.26	0.99

### Re-sequencing, data filter and sequence alignment

Genomic DNA of each bull was extracted from frozen sperms by a standard phenol-chloroform method [71]. DNA degradation and contamination were monitored on 1% agarose gels and the concentration and purity were assessed on NanoDrop 2000 (Thermo Scientific Inc. Waltham, DE, USA); the high-quality DNAs were then used for library construction. Two paired-end libraries were constructed for each individual, the read length was 2 × 100 bp, and whole genome sequencing was performed using Illumina Hiseq2000 instruments (Illumina Inc., San Diego, CA, USA). All processes were performed according to the standard manufacturer’s protocols. In order to get high-quality data, we removed low-quality reads and those containing primer/adaptor contamination which existing in the raw sequencing data by utilizing NGS QC Toolkit with default parameters [-l 75 -q 30] [72]. After data filtering, we used the Burrows-Wheeler Aligner (BWA) program [73] with default parameters [-A1 –B4] to perform sequence alignment based on the UMD3.1 genome assembly which was retrieved from the UCSC website (http://genome.ucsc.edu/). To save run time during the downstream analysis, we converted the SAM files to BAM files and then sorted and merged them by SAMtools [74].

### Detection of CNV

CNVnator was run on merged BAM files with a bin size of 200 bp following the authors’ recommendations [65]. After calling, quality control was performed on the raw CNVs for each bull. The filtering criteria included *P*-value <0.01 (pval1 calculated using *t*-test statistics), size >1 kb, and q0 < 0.5. *P*-value <0.01 means that the region between two calls is not a same CNV and q0 means fraction of mapped reads with zero quality. In addition, the CNVs that overlapped with gaps or unplaced chromosomes (chrUn in UCSC) were removed.

### Statistical analysis

According to the EBVs for PP and FP, the 8 Holstein bulls were divided into 2 groups, high-group and low-group, and the differential CNVs between the high and low groups were obtained as the following steps. Here, a differential CNVR describes a CNVR that was segregating within the two populations. Firstly, as for any two CNVs from any two individuals of 4 bulls within each group, they were considered to be common CNVs if they have >30% reciprocal overlap, then we obtained the common CNV regions (CNVRs) by merging the common CNVs across the four individuals in either the high or low groups, respectively. Secondly, after getting the common CNVRs in each group, differential CNVRs were identified between the high and low groups of bulls with extremely high/low PP and FP. To compare our results with previous studies, we used the UCSC liftOver tool [75] to convert the coordinates of CNVRs between UMD3.1 and Btau4.0.

### Quantitative PCR validation

Quantitative PCR (qPCR) was used to validate CNVRs detected by CNVnator. A total of 11 CNVRs was randomly chosen. For each CNVR, we firstly determined the best primers after designing multiple pairs of primers because of the uncertainty of the CNVR boundaries using Primer3 webtool (http://bioinfo.ut.ee/primer3-0.4.0/primer3/). To ensure the amplification efficiencies of all pairs of primers, a serial diluted genomic DNA sample from a common cattle was used as template for creating a standard curve of each pair of primer. The Basic Transcription Factor 3 *(BTF3)* gene was chosen as the control with the assumption that there were two copies of DNA segment in this region [69]. With a total volume of 15 μL reagents in a 96-well plate, qPCR was conducted using SYBR green chemistry in triplicate reactions on LightCycler® 480, Roche. The condition for thermal cycle was as follows: 5 min at 95 °C followed by 45 cycles at 95 °C for 10 s, 60 °C for 10 s and 72 °C for 15 s. The 2^-ΔΔCt^ method was used to calculate the relative copy number for each test region. First, we obtained the average Ct value of three replications of each sample and normalized against the control gene. Then we calculated the ΔCt value between the test sample and reference sample detected with normal status (i.e. two copy numbers) by CNVnator. Finally, a value around 3 or above was considered as gain and a value around 1 or below was considered as loss.

### Gene contents and functional annotation

Using the BioMart Database, the genes within the detected CNVRs were retrieved based on UMD3.1 sequence assembly (http://asia.ensembl.org/biomart/martview/). Ensembl genes overlapping with CNVRs completely or partially were considered as copy number variable and selected for further analysis. To provide insight into the functional enrichment of genes picked out above, we carried out annotation analysis, including GO (Gene Ontology) and KEGG (Kyoto Encyclopedia of Genes and Genomes), using KOBAS 2.0 [76], which annotates an input set of genes with putative pathways and disease relationships based on mapping to genes with known annotation. KOBAS 2.0 accepts ID and cross-species sequence similarity mapping and then performs statistical tests to identify statistically significantly enriched pathways and diseases. KOBAS 2.0 incorporates knowledge across 1327 species from 5 pathway databases (KEGG PATHWAY, PID, BioCyc, Reactome and Panther) and 5 human disease databases (OMIM, KEGG DISEASE, FunDO, GAD and NHGRI GWAS Catalog). All annotated Ensembl genes are used as background. In addition, we compared CNVRs with the reported cattle QTLs for milk PP and FP traits in the Animal QTL database [77].

## Results

### Sequencing data set statistics and CNV discovery

**Fig. 1:**
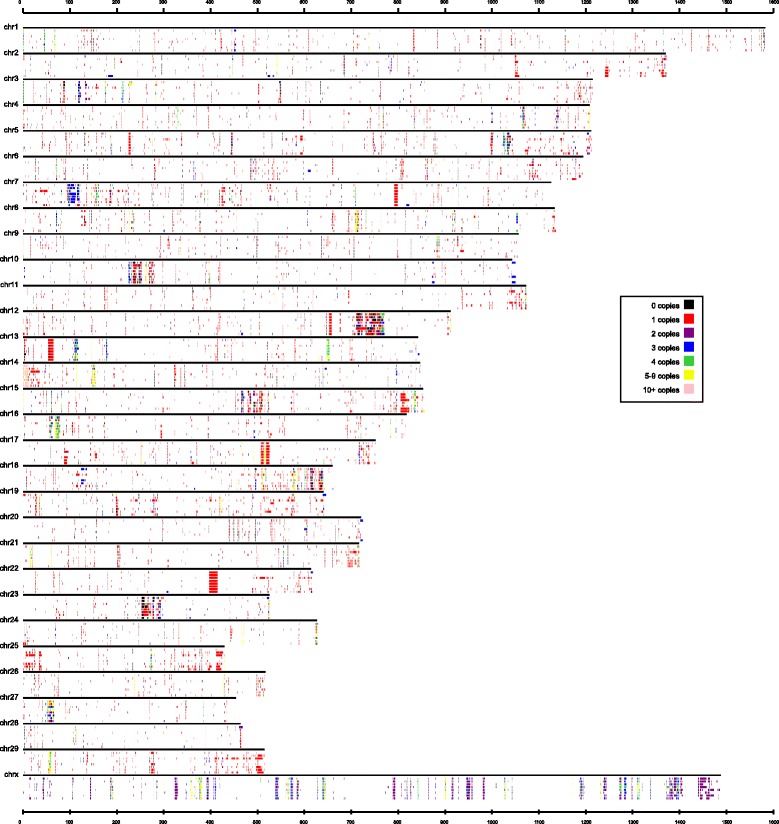
Individualized cattle CNV map. The tracks under every *black bar* represent the CNVs for sample 1 to sample 8 (in order from *top* to *bottom*). The *colors* for each *bar* in the animal data se tracks represent the average estimated CN for each CNV as shown in the legend

With Illumina paired-end sequencing technology, we obtained NGS data from the 8 Holstein bulls (Table 2). After we mapped them on the UMD3.1 bovine genome assembly and excluded potential PCR duplicates, the depth of coverage for each individual varied from 8.2× (sample 6) to 11.9× (sample 5). As shown previously, a 4x coverage is sufficient for CNV detection using a RD method [19, 23, 78]. With CNVnator, CNVs were detected for 8 individuals. After quality control, the number of duplication ranged from 687 (sample 6) to 777 (sample 4), and the number of deletion varied from 1091 (sample 1) to 1620 (sample 3) (Table 2). In order to determine how many CNVRs were detected from all 8 bulls, all the CNVs were merged if overlaps were 1 bp or greater, and a total of 6015 CNVRs were obtained. The detailed information about CNVs is shown in Additional file 1: Table S1. From Fig. 1, we can see the CNV landscapes roughly. Although chromosome 1 was the longest, the number of CNVs it contained was not the most in any individual. Chromosome X occupied the most CNVs and simultaneously the largest CNVs.

**Table 2:** Summary statistics of sequencing data and CNV of 8 Holstein bulls

No of bulls	Numbers of mapped reads	Depth	Duplication	Deletion
1	257615849	9.8	743	1091
2	246773374	9.4	708	1295
3	237335344	9.0	705	1620
4	252933841	9.6	777	1210
5	312273373	11.9	756	1348
6	215134987	8.2	687	1397
7	249257655	9.5	706	1373
8	251909753	9.6	705	1410

To confirm the CNVs detected by CNVnator, qPCR based on the relative comparative threshold cycle (C_T_) method was performed to validate 11 randomly chosen predicted CNVRs representing different types of duplication, deletion and both, on the same 8 samples for whole genome sequencing (Additional file 2: Table S2). It was shown that the validate rates of the 8 samples varied from 57.14% to 90% with an average of 73.04%.

### Identification of differential CNVRs between high and low groups

**Fig. 2:**
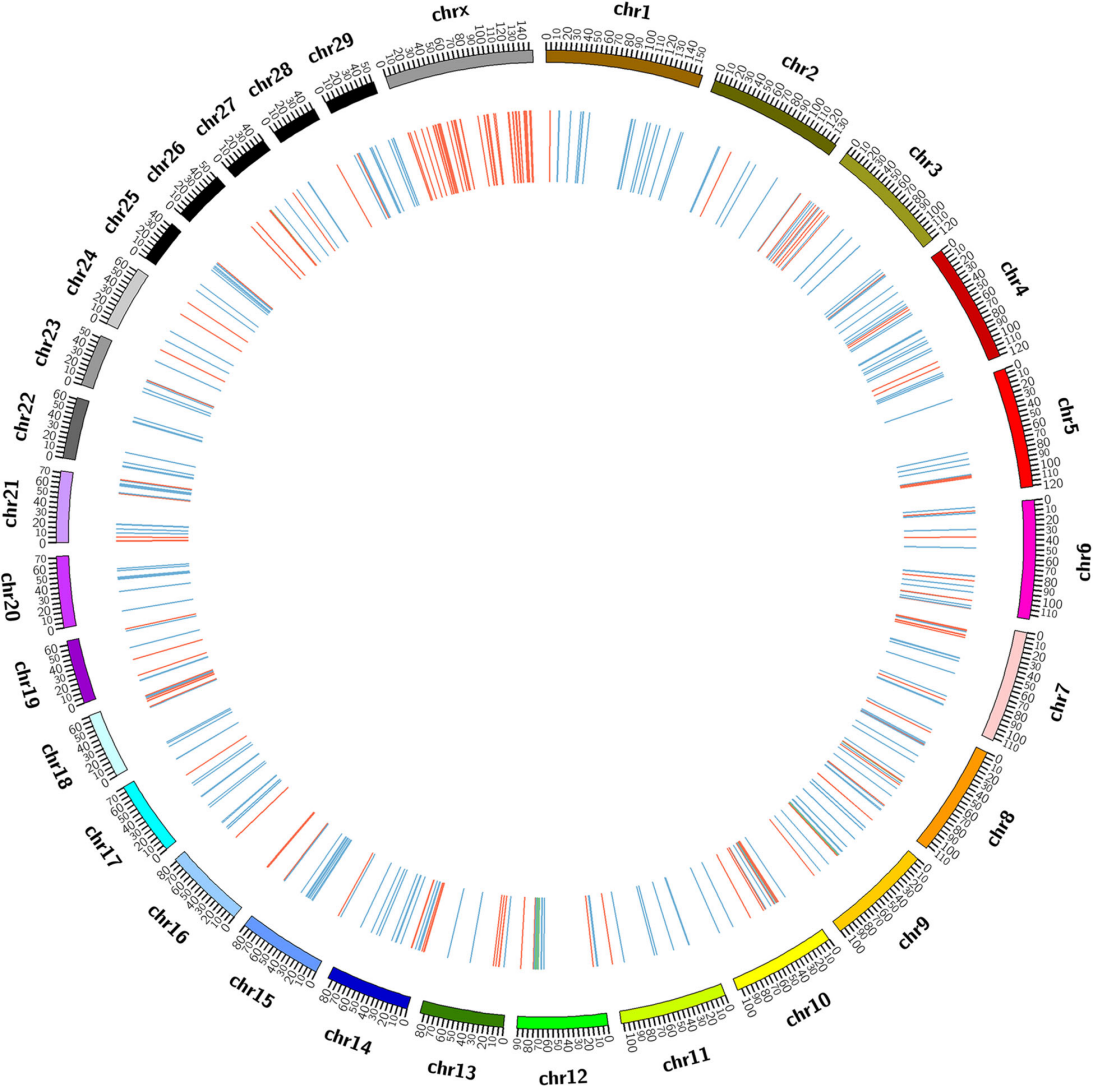
Different CNVRs between high and low group. Based on UMD3.1, 487 CNVRs derived from difference between high and low group were shown in *blue* (deletion), *red* (duplication), and *green* (both)

**Fig. 3:**
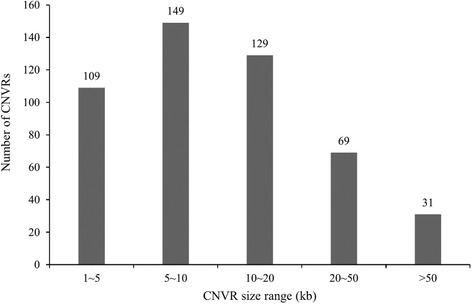
The length and frequency distribution of differential CNVRs

**Fig. 4:**
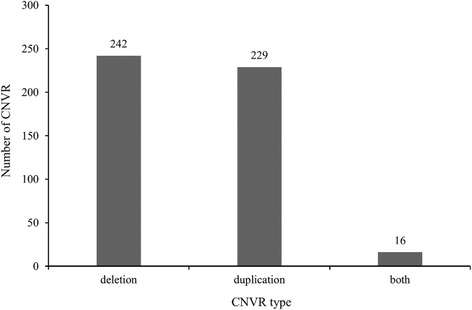
Types of differential CNVRs

According to the experimental design and filtering standards, we first screened common CNVRs shared by the high and low groups. Then these common CNVRs were excluded from whole CNVRs of high and low group respectively, and 268 and 280 CNVRs as group-specific in high and low group were remained. Finally, a total of 487 differential CNVRs were obtained after merging two group-specific CNVRs if overlaps were 1 bp or greater, covering chromosomes 1-X (Additional file 3: Table S3), which amounted to 8.23 Mb of the cattle genome (Fig. 2). The length of CNVRs varied from 1.6 kb to 275.6 kb with an average of 16.91 kb and a median of 9.4 kb (Table 3) and 31.3% of all CNVRs had sizes ranging from 5 kb to 10 kb (Fig. 3). The CNVRs were divided into 3 categories, i.e. 242 deletions, 229 duplications and 16 both events (Fig. 4). In terms of count and length, deletion and duplication CNVRs were almost similar (242 vs 229, 3.89 Mb vs 3.58 Mb).

**Table 3:** Characterization of cattle CNVs detected in different studies based on re-sequencing data

Studies	Summary statistics of CNVRs					
	Mean (Kb)	Median (Kb)	Min (Kb)	Max (Kb)	Std	Total (Mb)
Bickhart et al.	43.95	23.63	10.02	510.94	54.45	55.59
Zhan et al.	6.98	3.8	3.17	129.97	10.29	3.63
Stothard et al.	4.16	3.17	1.84	28.03	2.96	3.29
This study	12.47	7.2	1.2	422.8	19.82	72.02

### Gene contents of differential and group-specific CNVRs

#### Differential CNVRs

Utilizing BioMart in the Ensembl database (Ensembl Genes 79), we obtained the IDs for the genes that are located within or overlapped with the detected CNVRs. As a result, a total of 235 genes were identified, including 218 protein-coding genes, 5 miRNA genes, 4 snRNA, 3 pseudogenes, 3 rRNA and 2 snoRNA (Additional file 4: Table S4) and 29.98% of the CNVRs encompass 1 or more genes. To know about the biological functions of these genes, GO and KEGG pathway analysis were performed with KOBAS. We found that there were 163 significant GO terms and 8 significant KEGG pathways. GO terms related to protein and lipid metabolism were enriched (*p* < 0.05), such as long-chain fatty acid binding, protein glycosylation, asymmetric protein localization, glycoprotein biosynthetic process, protein serine/threonine kinase activator activity and negative regulation of protein acetylation. Also, the enriched KEGG pathways included several well-known protein and lipid metabolisms pathways (*p* < 0.05) such as insulin/IGF pathway-protein kinase B signaling cascade, prolactin signaling pathway and AMPK signaling pathway (Additional file 5: Table S5).

#### Group-specific CNVRs

Furthermore, we obtained 106 and 139 genes based on the 268 and 280 CNVRs across 4 individuals in the high and low groups, respectively. In high group, there were 2 significant GO terms, including lipid metabolism and glucose metabolic processes, and 1 significant KEGG pathways, a well-known lipid metabolisms pathways (prolactin signaling pathway) (*p* < 0.05) (Additional file 6: Table S6). In low group, 3 significant GO terms, i.e., dopamine biosynthetic process and insulin receptor binding, and 1 significant KEGG pathway (olfactory transduction) were enriched (*p* < 0.05) (Additional file 6: Table S6).

### Quantitative traits locus overlapped with differential and group-specific CNVRs

#### Differential CNVRS

We compared the detected differential CNVRs between high and low groups with the previously reported cattle QTL regions for milk production traits (cattle QTL database, http://www.animalgenome.org/cattle/) in order to further study the potential genetic effects of these CNVRs. Finally, 75 QTLs for protein yield, protein percentage, fat yield and fat percentage were found to be overlapped with 95 CNVRs (Additional file 7: Table S7), implying the functional genes within these CNVRs are likely candidates for milk protein and fat traits.

#### Group-specific CNVRs

In addition, we compared 268 CNVRs in the high group with cattle QTL regions for same traits as above. Totally, 46 QTLs were obtained which overlap with 40 CNVRs (Additional file 8: Table S8). Similarly, we compared 280 CNVRs with corresponding traits in the low group and 55 QTLs overlapped with 52 CNVRs were found (Additional file 8: Table S8).

## Discussion

In this study, we detected genome-wide CNVs of 8 Holstein bulls with extremely high and low EBVs for PP and FP based on NGS using CNVnator. We obtained 1834 ~ 2326 CNVs with an average of 2066.5 per bull. Compared with the previous methods based on SNP chip and aCGH of detecting CNV, NGS has many advantages in terms of both CNV numbers and sizes because the sequencing approach overcomes the sensitivity limits in the previous methods, and can more precisely identify CNV boundaries [79]. With the ongoing developments and cost decreases in NGS, the sequencing approaches has become more and more popular for CNV detection. Due to the fact that it was not designed for CNV detection specifically and incomplete coverage of the whole genome, SNP chip was restricted in the application of CNV detection.

**Fig. 5:**
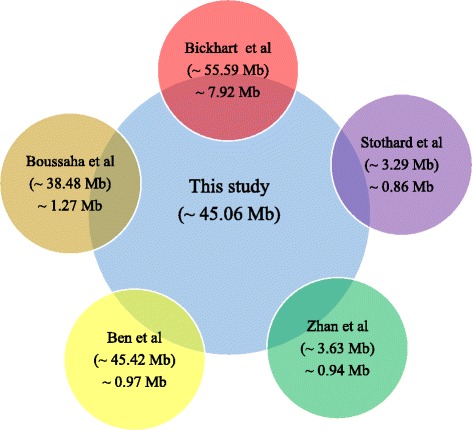
Comparison between 3996 CNVRs in this study and the other cattle CNVR datasets on Btau4.0

Based on the observation of CNV distribution, they were enriched in centromeric and the subtelomeric which is in agreement with the distribution of the cattle SD regions as reported before [80]. The number of CNVs (14,821) identified in this study was more than the reports based on NGS data by Bichkhart et al. (1265) in Angus, Holstein, Hereford and Nelore cattle [23], Stothard et al. (790) in Holstein and Black Angus [28], Zhan et al. (520) in Holstein [27], Boussaha et al. (957) in Holstein, Montbéliarde and Normande [29] and Ben et al. (823) in Holstein [30]. In addition, Jiang et al. detected CNVs based on Illumina BovineSNP50 (99) and BovineHD chips (367) data in Chinese Holstein population [24, 25], which were less than what we detected in this study. After converting 6015 CNVRs to corresponding results based on Btau4.0 using the UCSC liftOver tool with 50% of bases that must remap, 3996 CNVRs of which were successfully converted amounting to about 45.06 Mb. We found that ~80% of the 3996 CNVRs overlapped with those reported by previous investigations [23, 27–30] by 1 bp or greater (Fig. 5), and the largest overlap was ~7.92 Mb of the reported by Bickhart et al. [23]. As for the above inconsistencies, there are likely due to different detection methods and different samples. Bickhart et al. used mrFAST/mrsFAST and WSSD [23], and both Zhan et al. [27] and Stothard et al. [28] performed CNV-seq, and Boussaha et al. [29] used GATK, Pindel, and Ben et al. [30] performed control-FREE. While in this study, we used CNVnator. In addition, different cattle breed with specific genetic background may induce the inconsistencies of number of CNVs and CNVRs among various studies as well. In this study, the qPCR validation rates of the detected CNVs was 57.14% to 90%, which was similar to those reported by Bickhart et al. (82%) [23], Zhan et al. (86%) [27], Stothard et al. (100%) [28] and Yi et al. (91.7%) [41], showing the high accuracy of NGS-based CNV detection. The relatively lower validation rate in this study may be due to the following reasons: (1) false positive in CNV calling even if CNVnator has a low false-discovery rate (3% ~ 20%) [65], (2) primers in qPCR experiment were not the best although we tried multiple primers. As we know, there may be potential SNPs and small INDELs in the genome, and the negative impact of these potential variants could result in the reduced primer efficiency.

Genome wide CNV detection is also a strategy to identify the potential key genes for the traits of interest by mining the genes within the CNVRs in a specific experimental design. Hence, the different CNVRs between the high and low groups of Holstein bulls with extremely high and low EBVs for PP and FP were used for candidate gene identification for milk protein and milk fat. We determined a total of 487 differential CNVRs between the high and low groups, and further found that 235 functional genes were located within these CNVRs. Function analysis showed that the 235 genes were enriched in 163 significant GO terms and 8 significant KEGG pathways. Especially, the processes of long-chain fatty acid binding, protein glycosylation, asymmetric protein localization, glycoprotein biosynthetic process, protein serine/threonine kinase activator activity and negative regulation of protein acetylation were related to protein or lipid metabolism were enriched. Also among the KEGG pathways we detected, insulin/IGF pathway-protein kinase B signaling cascade, prolactin signaling pathway and AMPK signaling pathway are well-known pathways for protein and lipid metabolism [81, 82] and 10 genes involved in the biological process such as cell apoptosis, protein modification, conversion of amino acid and metabolism of fatty acids were included. These were *INS, IGF2, FOXO3, TH*, *SCD5, GALNT18, GALNT16, ART3*, *SNCA* and *WNT7A.*

The bovine insulin (*INS*) gene is close to the *IGF2* and *TH* genes. Insulin binding to the insulin receptor (INSR) exerts biological function that maintaining the blood glucose concentration through multiple signaling pathways, such as AMPK, insulin, mTOR and PI3K-Akt signaling pathways, which play critical roles in milk fat and protein synthesis in dairy cattle [83]. The *IGF2* gene was related to breast epithelial and stromal cell proliferation in human [84], and over-expression of *IGF2* increased breast cancer development [85], thus, it was implied that *IGF2* may play an important role in maintaining bovine mammary gland epithelial cell function well. Forkhead box O3 (*FOXO3*) was also known as *FOXO3a*, which was considered as a key downstream effector of the well-known signaling pathways for lipid and protein metabolism, i.e. PI3K-Akt, MAPK and Jak-STAT [86, 87]. Tyrosine hydroxylase (TH) is the rate limiting enzyme for converting tyrosine to dopamine which was a crucial regulator of prolactin (PRL) [88]. PRL is essential for mammary gland involution and lactation [88, 89]. The Stearoyl-CoA desaturase 5 (*SCD5*) gene is located within a known QTL region for milk protein [90] and fat yield [91], but also near to the SNPs significantly associated with milk fat yield, protein yield, fat percentage and protein percentage identified by a previous GWA study [15]. The ADP-ribosyltransferase 3 (*ART3*) gene encodes the arginine-specific ADP-ribosyltransferase that has impact on cell proliferation and apoptosis etc. [92]. The Wnt family member 7A (*WNT7A*) gene, as a member of WNT gene family, encodes secreted signaling proteins and is related to suppressing human lung cancer progression [93]. The synuclein alpha (*SNCA*) gene was found to be associated with Parkinson’s and Alzheimer’s diseases [94, 95]. Among the candidate genes, *INS, IGF2, FOXO3, TH*, *SCD5* were related with milk composition traits according to previous studies, and identification of them in current study confirmed their potential functions. As for the remaining genes, there existed more or less indirect association with milk composition traits. Thereby, to gain further insights into the association of the 10 candidate genes with milk composition traits, we compared the chromosome positions of the 10 genes with the significant SNPs detected by previous GWAS for milk production traits in dairy cattle [4, 5, 7, 10, 15], and found that all genes were located near to multiple significant SNPs for milk protein and fat traits with 0.01 Mb to 9.90 Mb (Additional file 9: Table S9), suggesting their potential associations with milk compositions.

In the study of Xu et al. [13], 34 CNVs were found significantly associated with milk production traits, of which 11 CNVs were included in the differential CNVRs identified in this study, i.e., CNVR45, CNVR46, CNVR47, CNVR189, CNVR190, CNVR200, CNVR201, CNVR202, CNVR203, CNVR399 and CVNR400. Within CNVR400, two candidate genes, *INS* and *IGF2* were enriched. Ben et al. [30] identified two CNVRs associated with milk composition, including one (chr17: 75031000–75158596) with milk fat yield and milk protein yield, and another (chr18: 12381000–12527000) with milk fat yield, and 8 genes were enriched in these two regions, especially the *MTHFSD* gene within the second CNVR belongs to the folate metabolism gene family and plays critical roles in regulating milk protein synthesis [96]. Although there was no overlap between these CNVRs and ours, two CNVRs in this study were located near to them with 2.51 Mb and 5.83 Mb, respectively. The *DEPDC5* gene overlapped with CNVR290 encodes a protein which was a component of the GAP activity toward Rags complex and is involved in mTORC1 pathway [97].

In addition, we found that 95 differential CNVRs detected in this study were overlapped with 75 known QTLs that have been shown to be associated with protein yield, protein percentage, fat yield and fat percentage in dairy cattle (Cattle QTLdb, http://www.animalgenome.org/cgi-bin/QTLdb/BT/index). Eight annotated genes were overlapped with these differential CNVRs (Additional file 7: Table S7).

## Conclusions

In conclusion, based on NGS data of 8 Holstein bulls with extremely high and low EBVs for milk PP and FP, we identified a total of 14821 CNVs corresponding to 6015 CNVRs. Of these, 487 differential CNVRs between the high and low groups were obtained. Of note, we further identified 235 annotated genes that were located in or overlapped with these differential CNVRs, including 10 genes significantly enriched for specific biological functions related to protein and lipid metabolism, insulin/IGF pathway-protein kinase B signaling cascade, prolactin signaling pathway and AMPK signaling pathways. These genes included *INS, IGF2, FOXO3, TH, SCD5, GALNT18, GALNT16, ART3, SNCA* and *WNT7A*, implying their potential association with milk protein and fat traits.

## Additional files

Additional file 1: Table S1. Summary of identified CNVs and CNVRs in the 8 bulls’ genomes. (XLSX 1044 kb)

Additional file 2: Table S2. Primers information and confirmation results of the 11 chosen CNVRs by qPCR analysis. (XLSX 14 kb)

Additional file 3: Table S3. General statistics of 487 differential CNVRs between high and low group based on UMD3.1. (XLSX 28 kb)

Additional file 4: Table S4. The detailed features of genes completely or partial overlapped with differential CNVRs. (XLSX 28 kb)

Additional file 5: Table S5. Functional enrichment of GO and KEGG pathway analysis of genes covered by differential CNVRs. (XLSX 17 kb)

Additional file 6: Table S6. Functional enrichment of GO and KEGG pathway analysis of genes covered by group-specific CNVRs. (XLSX 11 kb)

Additional file 7: Table S7. QTLs covered by differential CNVRs. (XLSX 18 kb)

Additional file 8: Table S8. QTLs covered by group-specific CNVRs. (XLSX 17 kb)

Additional file 9: Table S9. Detailed information on the significant SNPs identified in previous GWAS that are near to the 10 candidate genes identified in this study. (XLSX 17 kb)

## Abbreviations

Acgh: Array comparative genomic hybridization
AS: Assembly
BTF3: Basic transcription factor 3
BWA: Burrows-wheeler aligner
CN: Copy number
CNV: Copy number variation
CNVR: CNV region
EBV: Estimated breeding value
FoSTeS: Fork stalling and template switching
FP: Fat percentage
GO: Gene ontology
GWAS: Genome-wide association analysis
KEGG: Kyoto encyclopedia of genes and genomes
LA: Linkage analysis
NAHR: Non-allelic homologous recombination
NGS: Next-generation sequencing
NHEJ: Non-homologous end joining
PP: Protein percentage
qPCR: Quantitative PCR
QTL: Quantitative traits locus
RD: Read-depth
RP: Read pair
SD: Segmental duplications
SNP: Single nucleotide polymorphisms
SR: Split-read
